# Supplementary material for: The interactome of intact mitochondria by cross-linking mass spectrometry provides evidence for coexisting respiratory supercomplexes
Source: Mol Cell Proteomics. 2017 Dec 8;17(2):216–32. doi: 10.1074/mcp.RA117.000470 (PMC5795388; doi:10.1074/mcp.RA117.000470)
Supplement: Supplemental Data [file supp_RA117.000470_133922_0_supp_23978_fzffwf.zip › spectra_annotation/mito_spectra_annotation/348-1-3-1-6-1.pdf]

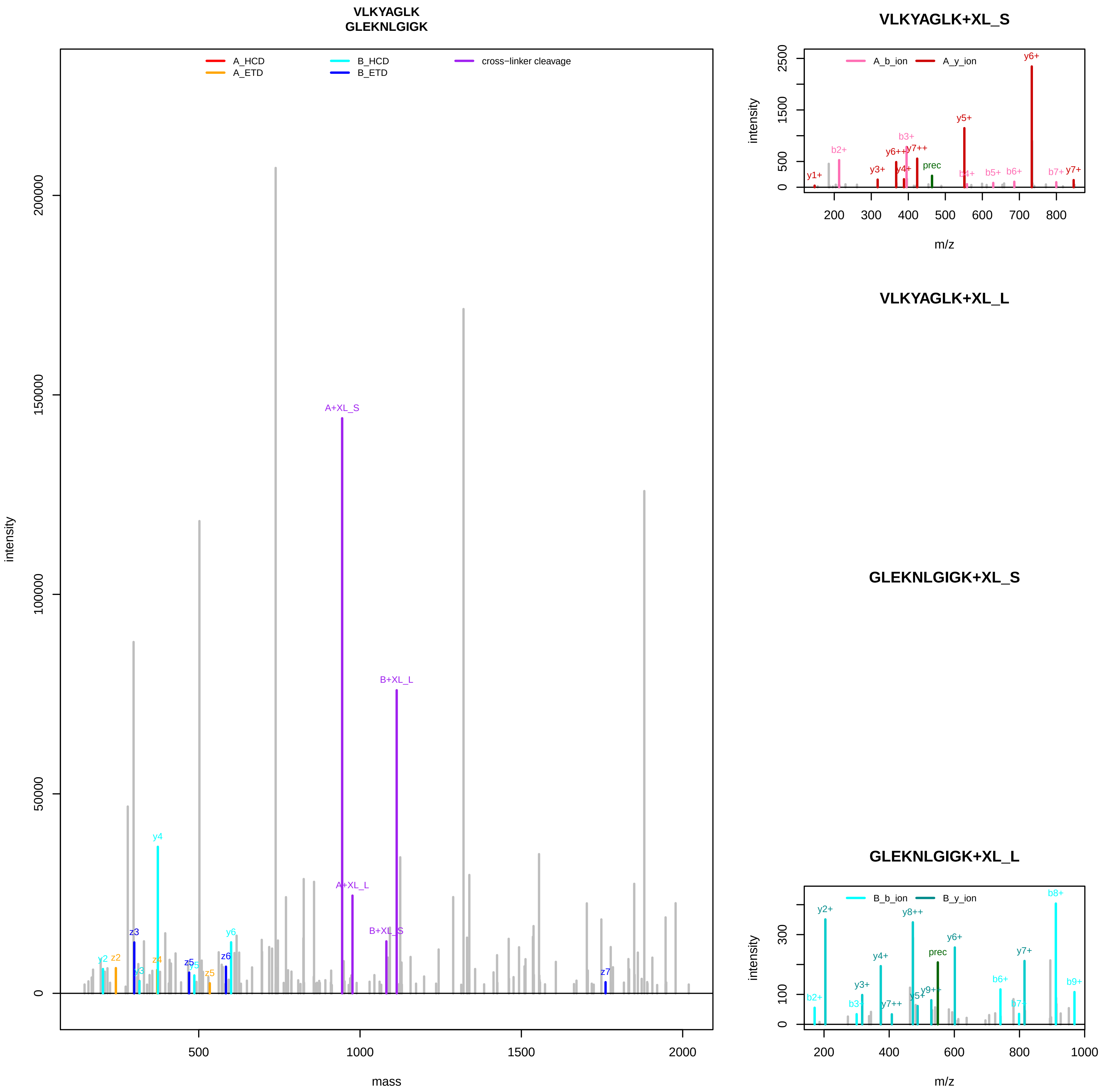

VLKYAGLK
GLEKNLGIGK
A_HCD
A_ETD
B_HCD
B_ETD
cross−linker cleavage
A+XL_S
B+XL_L
A+XL_L
B+XL_S
y2
z2
z3
y3
y4
z4
z5
y5
z5
z6
y6
z7
intensity
mass
VLKYAGLK+XL_S
A_b_ion
A_y_ion
y1+
b2+
y3+
y6++
y4+
b3+
y7++
prec
y5+
b4+
b5+
b6+
y6+
b7+
y7+
m/z
VLKYAGLK+XL_L
GLEKNLGIGK+XL_S
GLEKNLGIGK+XL_L
B_b_ion
B_y_ion
b2+
y2+
b3+
y3+
y4+
y7++
y8++
y5+
y9++
prec
y6+
b6+
b7+
y7+
b8+
b9+
